# Effects of a multi-professional intervention on body composition, physical fitness and biochemical markers in overweight COVID-19 survivors: a clinical trial

**DOI:** 10.3389/fphys.2023.1219252

**Published:** 2023-08-28

**Authors:** Ana Flávia Sordi, Maurício Medeiros Lemos, Déborah Cristina de Souza Marques, Joed Jacinto Ryal, Marielle Priscila de Paula Silva Lalucci, Marilene Guiraldi Marques, Maria Luiza Amaro Camilo, Solange De Paula Ramos, Solange Marta Franzói De Moraes, Pablo Valdés-Badilla, Jorge Mota, Braulio Henrique Magnani Branco

**Affiliations:** ^1^ Interdisciplinary Laboratory of Intervention in Health Promotion, Cesumar Institute of Science, Technology and Innovation, Maringá, Brazil; ^2^ Graduate Program in Health Promotion, Cesumar University, Maringá, Brazil; ^3^ Graduate Program of Physical Education, State University of Londrina, Londrina, Brazil; ^4^ Graduate Program of Physiology, State University of Maringá, Maringá, Brazil; ^5^ Department of Physical Activity Sciences, Faculty of Education Sciences, Universidad Católica del Maule, Talca, Chile; ^6^ Sports Coach Career, School of Education, Universidad Viña del Mar, Viña del Mar, Chile; ^7^ Laboratory for Integrative and Translational Research in Population Health (ITR), Research Centre of Physical Activity, Health, and Leisure, Faculty of Sports, University of Porto, Porto, Portugal

**Keywords:** coronavirus, health promotion, multi-professional intervention, physical exercise, chronical disease

## Abstract

**Introduction:** The sequelae post-COVID can affect different systems. In this sense, considering the multi-factorial etiology of COVID-19, multi-professional interventions could be a relevant strategy for recovery health indicators.

**Objective:** This study aimed to investigate the effects of multi-professional intervention on body composition, physical fitness, and biomarkers in overweight COVID-19 survivors with different symptomatology.

**Methodology:** A non-randomized parallel group intervention included 69 volunteers (BMI ≥25 kg/m^2^), divided into three groups according to SARS CoV-2 symptomatology, but only 35 finished the longitudinal protocol [control group (*n* = 11); moderate group (*n* = 17) and severe group (*n* = 7)]. The groups were submitted to a multi-professional program (nutritional intervention, psychoeducation, and physical exercise intervention) for 8 weeks, and the volunteers underwent body composition assessments (primary outcome) and physical and biochemical tests (secondary outcome) in pre- and post-intervention. This study was registered on the Clinical Trials Registration Platform number: RBR-4mxg57b and with the local research ethics committee protocol under number: 4,546,726/2021.

**Results:** After the 8-week multi-professional intervention, the following results were observed for the moderate COVID-19 group: improved dynamic strength of lower- and (*p* = 0.003), upper-limbs (*p* = 0.008), maximal isometric lumbar-traction strength (*p* = 0.04), flexibility (*p* = 0.0006), and albumin (*p* = 0.0005), as well as a reduction in the C reactive protein (CRP) (*p* = 0.003) and fasting glucose (*p* = 0.001); for the severe COVID-19 group: an improvement in dynamic lower-body strength (*p* = 0.001), higher values of albumin (*p* = 0.005) and HDL-c (*p* = 0.002), and lower values of CRP (*p* = 0.05), and for the control group: an improvement in sit-up repetitions (*p* = 0.008), and a reduction of CRP (*p* = 0.01), fasting glucose (*p* = 0.001) and total cholesterol (*p* = 0.04) were identified. All experimental groups reduced triglycerides after intervention (*p* < 0.05).

**Conclusion:** Finally, 8 weeks of multiprofessional intervention can be an efficient tool for reversing the inflammatory process and promoting improvements in daily activities and quality of life, although it is believed that the severe COVID-19 group needs longer interventions to improve different health indicators.

**Clinical Trial Registration:** https://ensaiosclinicos.gov.br/, identifier: RBR-4mxg57b.

## 1 Introduction

Long COVID-19, associated with several health problems due to sequelae of SARS-CoV-2 (Davis et al., 2023), is characterized by persistent post-COVID symptoms of 12 weeks or more (Astin et al., 2023). The sequelae can affect different systems, such as cardiopulmonary, immunological, neurological, skeletal muscle, circulatory, and mental health (Davis et al., 2023). To clarify the nature and frequency of persistent symptoms, *Van-Kessel et al.* (Van Kessel et al., 2022) concluded that fatigue, dyspnea, chest pain, and headache are long-COVID-19 affecting work and daily functioning. Thus, rehabilitation strategies for COVID-19 survivors are indispensable to combat a condition with different sequelae with significant impacts on the population, the health of individuals, and the economy (Astin et al., 2023). It is well established that obesity, comorbidities, and low physical activity levels may worsen the clinical outcome of COVID-19 survivors (Lemos et al., 2022). The World Health Organization (WHO) has published the *“Clinical Management of COVID-19: living guidance”* (World Health Organization, 2021a), establishing four classifications of COVID-19 symptoms, such as mild, moderate, severe, and critical, according to the progression and worsening of the symptoms of COVID-19. Symptom progression commonly depends on the primary health condition of the individual and the immune response provoked by the infection (Shi et al., 2020).

In this sense, answering questions regarding cardiorespiratory and neuromuscular treatment strategies can provide patients with a return to activities of daily living and consequent patient health improvement for those with long COVID (Lemos et al., 2022). Psychological sequelae have also been described in a previous study as factors that justify early multi-professional actions to recover the physical and mental health of COVID-19 survivors (Ryal et al., 2023). Although strategies to treat acute sequelae are under assessment, little attention has been given to the treatment of sequelae affecting the long-term quality of life. Therefore, interventions aimed at the patient’s recovery from actions that provide physical activity practice, healthy nutrition, and psychoeducation can reduce the sequelae of the disease and the complications resulting from the post-COVID syndrome in overweight individuals (Lemos et al., 2022).

Previous findings have investigated the role of physical exercise as a potential strategy to counteract the deleterious effects of COVID-19 but have yet to consider the other multi-professional aspects that involve public health promotion policies and the severity of COVID-19 (Dalbosco-Salas et al., 2021; Jimeno-Almazán et al., 2023). To the best of these authors’ knowledge, the effects of physical exercise, nutritional education, and psychoeducation, i.e., multi-professional interventions considering the specific symptoms (moderate and severe/critical) and including a control group (without diagnoses of COVID-19), were not investigated in the scientific literature. Considering the symptoms among the groups could be relevant to promote assertive interventions and recovery prognoses.

Therefore, the present study aimed to analyze the effects of multi-professional intervention on body composition, physical fitness, and biomarkers in overweight COVID-19 survivors. Based on previous studies (Dalbosco-Salas et al., 2021; Jimeno-Almazán et al., 2023), as a primary outcome, the authors of this research propose that the 8 weeks of multi-professional intervention model can improve body composition and physical fitness and, as a secondary outcome, metabolic parameters, regardless of the symptomatology of the disease.

## 2 Methods

### 2.1 Experimental approach to the problem

This study adopted an experimental design of repeated measures and parallel groups non-randomized for 8 weeks, following *Consolidated Standards of Reporting Trials* (CONSORT) (Schulz et al., 2010) from October to December 2021. The experimental groups (severe COVID-19, moderate COVID-19 group, and non-COVID-19 group/control group) were submitted to a multi-professional program of theoretical (nutritional intervention and psychoeducation) and physical exercise (concurrent training). Participants were assessed at baseline (pre-intervention) and after 8 weeks (post-intervention).

### 2.2 Participants

Participants were recruited via the Municipal Secretary of Health of Maringa, the Municipal Hospital of Maringa, and through TV, radio, and social media dissemination. The control group (non-COVID-19 group) was recruited through TV, radio, and social media dissemination. Interested parties contacted the Interdisciplinary Laboratory for Intervention in Health Promotion (LIIPS) multi-professional team at Cesumar University. Eighty-nine volunteers of both sexes were invited to participate in the study according to the following inclusion criteria: i) male and female participants between 19 and 65 years of age; ii) body mass index (BMI) > 25.0 kg/m^2^; iii) positive diagnosis confirmed via RT‒PCR (reverse transcriptase-polymerase chain reaction) for COVID-19 (only for moderate and severe COVID-19 groups); iv) received medical clearance to participate in the present study; v) received the first dose of COVID-19 vaccine; vi) available to participate in multi-professional interventions 2x/week for 8 weeks and vii) having contracted COVID-19 between January 03rd/2021 and July 01st/2021 (only for moderate and severe COVID-19 groups). An equivalent control group was recruited without COVID-19 diagnostics. Exclusion criteria included the following: i) debilitating neurological diseases (i.e., Alzheimer’s or Parkinson’s); ii) contraindications for physical exercise, and iii) pregnancy. Data collection and intervention occurred at the LIIPS in Maringa, Paraná, Brazil.

The *a priori* sample size calculation was based on weight loss (4.4 ± 4.0 kg) in 8 weeks of aerobic and resistance training program under dietary control in overweight men (Perissiou et al., 2020). Nine participants per group were necessary to achieve a statistical power of 80% with an alpha error of 5%. Since a large number (55.4%) of COVID-19 survivors have been reported to abandon exercise programs (Dalbosco-Salas et al., 2021), a minimum of 14 patients should be recruited for each study group. The present study was approved by the Local Research Ethics Committee (protocol n° 4,546,726) and followed the Declaration of Helsinki. The study was registered in the Brazilian Clinical Trials Registry Platform (REBEC) under RBR-4mxg57b. All subjects were informed about the purposes of the study and signed an informed consent form.

### 2.3 Procedures

Seventy-one volunteers were accepted to participate in the program and were allocated according to COVID-19 symptomatology (moderate or severe/critical symptoms) (World Health Organization, 2021b) and the control group (without the diagnosis of COVID-19), following the groups: severe COVID-19 (*n =* 16), moderate COVID-19 (*n =* 23), and control (*n =* 30). The baseline measures were conducted over 2 days. First, the subjects underwent a clinical assessment by a pulmonologist and an ICU physician, consisting of patient history (history of surgeries, preexisting non-communicable chronic diseases, continuous use of medications, main signs and symptoms presenting possible sequelae of COVID-19, and type and length of stay at the hospital (ward/room or intensive care unit)), anthropometric and body composition assessment and blood collection for biochemical analyses.

On the second day, the following data were collected: i) blood pressure (BP) after 5 min of rest, according to the VIII Guideline on Arterial Hypertension (Barroso et al., 2020); ii) measurement of heart rate (HR) and peripherical oxygen saturation (%SpO_2_), both at rest; iii) posterior chain flexibility test on the Wells bench (sit and reach test); iv) maximum isometric handgrip strength (MIHS) and maximum isometric lumbar traction (MILT) with specific dynamometers; v) sit-up test; vi) 30-s chair-stand-test; vii) push-up and (viii) cardiorespiratory fitness test [6-min walk test (6MWT)]. After the 6MWT, the following variables were collected: BP, HR, and %SpO_2_. All tests are described in the sections below. After the clinical assessment, the self-reported signs and symptoms were considered for the non-randomized allocation of participants in the experimental COVID-19 groups according to the *“Clinical Management of COVID-19: living guidance”* (World Health Organization, 2021a).

**FIGURE 1:**
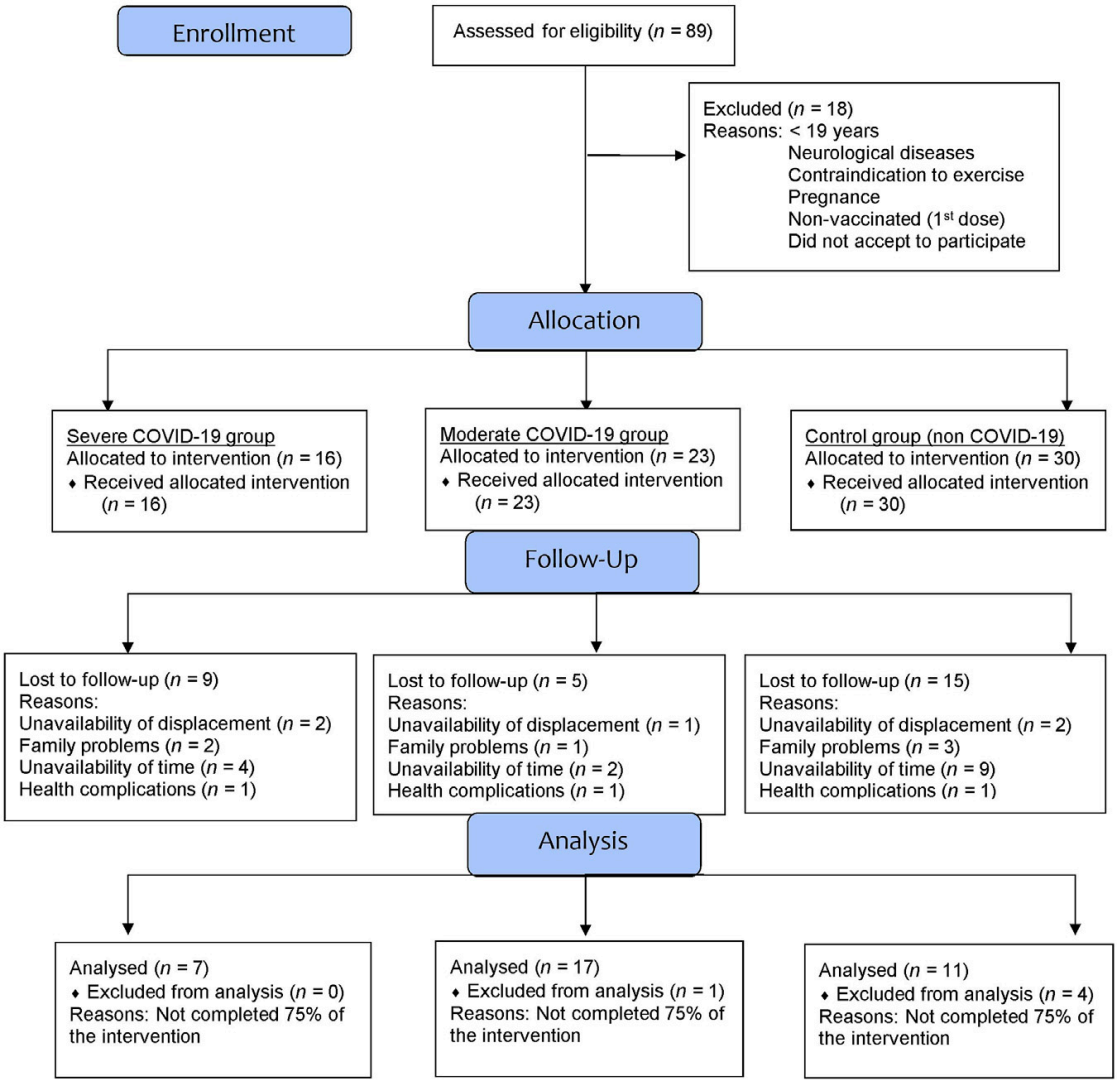
Flowchart diagram of the participants of the present study.

**FIGURE 2:**
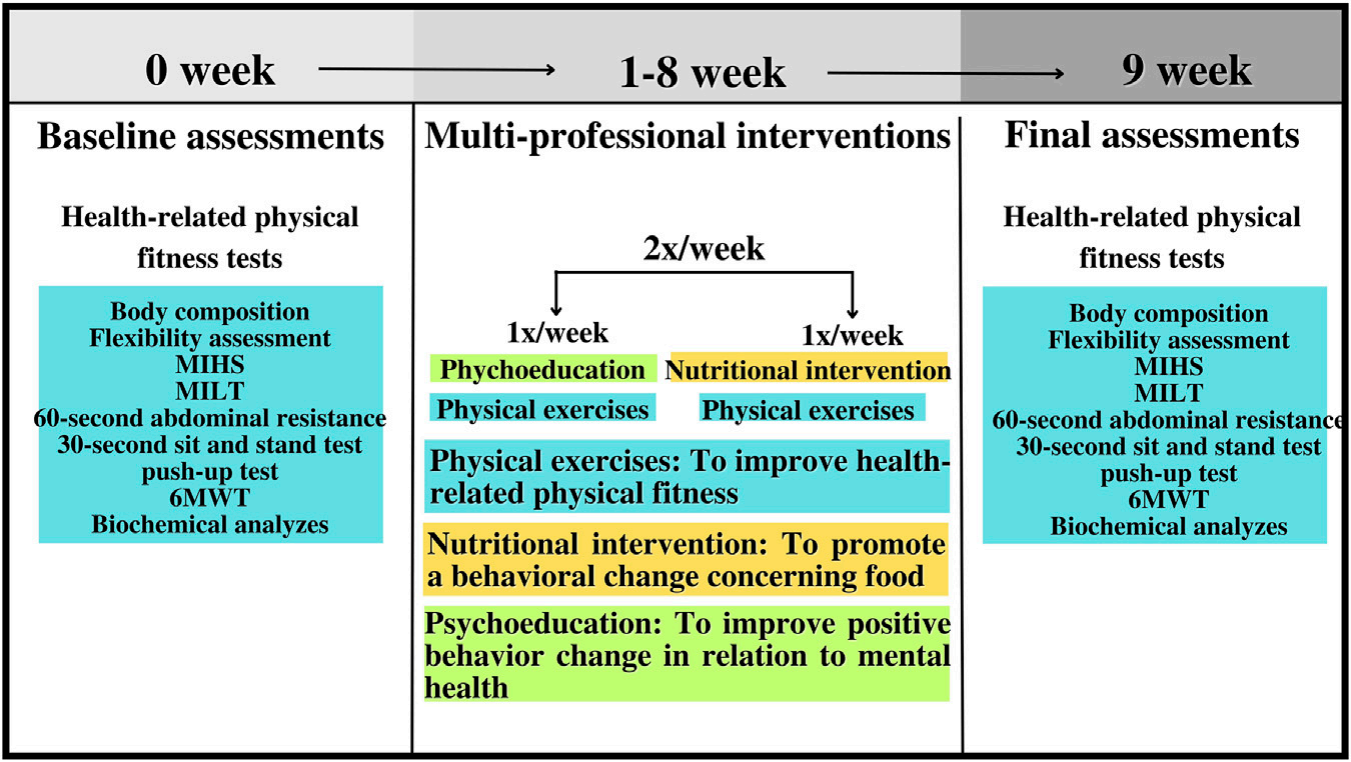
Methodological design of the present study. Note: MIHS = maximal isometric handgrip strength; MILT = maximal isometric lumbar-traction strength; 6MWT = 6-min walk test.

Over the 8 weeks, 25 participants dropped out of the project for different reasons. Figure 1 presents the flowchart of the present study’s participants based on the CONSORT Guidelines (Schulz et al., 2010) and Figure 2 illustrates the methodology used for the 8-week intervention.

### 2.4 Body composition

Participants’ height was measured using a stadiometer (Welmy R-110^®^, Santa Bárbara D′ Oeste, São Paulo, Brazil) coupled to a scale with 2.2 m capacity and 0.1 cm accuracy. Participants’ body composition was measured using tetrapolar bioimpedance (*InBody* 570^®^, *Biospace* Co. Ltd., Seoul, Korea), with a capacity of 250 kg and accuracy of 100 g, according to the manufacturer’s instructions and following the recommendations to improve the validity and reliability (Heyward, 2001). All participants were previously instructed about the recommendations. The following parameters were measured: BMI (kg/m^2^), lean mass (kg), fat mass (kg), body fat percentage (%), and skeletal muscle mass (kg).

### 2.5 Health-related physical fitness tests

The chosen physical tests to evaluate the outcomes of the COVID-19 survivors follow the order: i) sit and reach test; ii) maximum isometric handgrip strength (MIHS), maximum isometric lumbar traction (MILT); iii) sit-up test for abdominal strength-resistance; iv) 30-s chair-stand-test for lower limbs; v) push-up test for upper limbs and (viii) cardiorespiratory fitness test [6-min walk test (6MWT)]. The participants were instructed about the procedures for all physical tests, and the researchers respected a rest between the tests. Furthermore, the choice of physical tests was based on promoting the assessment of physical fitness test parameters in places with low resources and clinics, public hospitals, gyms, and others.

### 2.6 Flexibility assessment

The sit and reach test was employed to evaluate the flexibility of the posterior chain using the Wells Bench. Participants were instructed according to previously described procedures (Wells and Dillon, 1952). The test was repeated three times, with a 60-s interval between attempts. The highest value obtained was recorded and expressed in cm.

### 2.7 Maximal isometric strength tests

To assess MIHS, a TKK 5101 dynamometer (Takei Physical Fitness Test^®^, Tokyo, Japan) with a capacity of 100 kg was used. MILT was evaluated using a Takei dynamometer (Takei Physical Fitness Test^®^, Back Strength Dynamometer, type 2, Japan) with a capacity of 300 kg. According to previous recommendations, three trials were performed for both tests, lasting 3–5 s with a 1-min rest between trials (Branco et al., 2018). The highest value was recorded in kg.

### 2.8 Dynamic muscle strength-endurance assessment

To assess dynamic muscle, sit-up, 30-s chair-stand, and push-up tests were performed according to the procedures described in previous studies (Jones et al., 1999; Cuenca-Garcia et al., 2022). For the sit-up and push-up tests, the maximum number of repetitions achieved in 60 s was recorded, and for the 30-s chair-stand test, the muscular endurance of the lower limbs was evaluated from the maximum number of repetitions performed in 30 s.

### 2.9 Cardiorespiratory fitness test

The 6MWT was applied to verify the cardiorespiratory fitness of the present study participants. The 6MWT test was performed per American Thoracic Society guidelines (ATS Committee on Proficiency Standards for Clinical Pulmonary Function Laboratories, 2002). Volunteers were instructed to walk as fast as possible to achieve the greatest distance at the end of 6 min (ATS Committee on Proficiency Standards for Clinical Pulmonary Function Laboratories, 2002). The peak oxygen consumption (VO_2_peak) was calculated using a previous study (Cahalin et al., 1996).

### 2.10 Biochemical analyses

The blood collection procedures followed the guidelines of the Clinical and Laboratory Standards Institute (Williamson and Synder, 2013). Participants were previously instructed on how to prepare for the collections that took place at the Clinical Analysis Laboratory of the University facilities, and after collection, participants were instructed to press on the puncture site to avoid bruising. The collected blood samples were distributed in the following tubes: Vacuplast^®^ collection tubes, tubes with the anticoagulant ethylene diamine tetra acetic acid (EDTA) K2, and tubes with anticoagulant fluoride/EDTA. Subsequently, to obtain serum and plasma, the samples containing the fluoride/EDTA activator were centrifuged in a CENTRILAB^®^ analog centrifuge at 3,500 rpm (relative centrifugal force) for 15 min at room temperature. The following laboratory tests were analyzed: glycemic control, lipid profile (total cholesterol, HDL-c: high-density lipoprotein, LDL-c: low-density lipoprotein, and TGL: triglycerides), liver enzymes (ALT: alanine aminotransferase, AST: aspartate aminotransferase, ALP: alkaline phosphatase, GAMMA-GT: gamma-glutamyl transferase and albumin), C-reactive protein (CRP) and glycated hemoglobin (HbA1C). The analyses were performed using Gold Analisa Diagnostic Kits (Belo Horizonte, Minas Gerais, Brazil) in the semiautomatic biochemical and turbidimetric analyzer device URIT 8021^®^ from MHLab. All analyses were performed in triplicate. The Finecare ^®^ FIA Meter Plus analyzer from WONDFO was used for HbA1C.

### 2.11 Physical exercise intervention

The physical exercise intervention sessions lasted approximately 60 min and were held in the university facilities. Physical exercises focused on improving cardiorespiratory and neuromuscular fitness (concurrent training) to increase muscle strength and, if necessary, motor coordination and balance. The concurrent training plan consisted of performing 2 weeks of anatomical adaptation with low volume and intensity, that is, 3 sets of 15 repetitions and 5 min of aerobic exercise at the end of the session, and the other weeks of physical exercise (plus 6, in total) had volume progression and gradual intensity (via classic linear model); that is, the loads used were readjusted over the weeks, as well as the number of sets and repetitions. In weeks 3 and 4, 3 sets of 12 repetitions were performed; in weeks 5 and 6, the training sessions consisted of 3 sets of 20 repetitions; and finally, in weeks 7 and 8, 4 sets of 12 repetitions were performed. Concerning aerobic exercise, 2 sets of 5 min were performed in weeks 3 and 4; 1 set of 5 and 1 set of 10 min was performed in weeks 5 and 6; and finally, in weeks 7 and 8, 2 sets of 10 min were performed. The concurrent training was performed twice weekly, with resistance exercises focused on large muscle groups and cardiorespiratory fitness performed on a treadmill, vertical/horizontal bicycle, or rowing ergometer, according to the preference and physical condition of the volunteers. For a third day, the participants were requested to improve their physical activity (especially walking, 1 hour a week–if possible, following their physical training). Table 1 presents the training program performed by the experimental groups during the 8 weeks of multi-professional intervention.

**TABLE 1:** The training program for the severe COVID-19*,* moderate COVID-19*,* and control groups.

Order	Training program a	Training program B
1	Warm-up	Warm-up
2	Plank torso strength	Plank torso strength
3	Rectus abdominis	Rectus abdominis
4	Aerobic exercises (5′)	Hip bridge
5	Squat	Leg press
6	Leg extension	Aerobic exercises (5′)
7	Bench press	Leg curl
8	Aerobic exercises (5′)	Push up
9	Cable pulldown	Cable straight back seated row
10	Dumbbell shoulder press	Front raise
11	Triceps pulley	Biceps curl
12	Aerobic exercises (5′)	Aerobic exercises (5′)

### 2.12 Training monitoring

The concurrent training sessions were monitored via perceptual scales (rating of perceived recovery and exertion). Before each training session, the perceived recovery status (RPR) scale proposed by *Laurent et al.* (Laurent et al., 2011), which identifies the recovery status, was used. After the end of the training session, the rating of perceived exertion (RPE) to quantify the intensity of the training session, proposed by *Foster et al.* (Foster et al., 2001), was measured. All volunteers were instructed about the scales in a meeting before starting the physical exercises. SpO_2_ and blood pressure (systolic: SBP and diastolic: DBP) were measured before (initial) and after (final) each exercise session. In addition, the volunteers self-reported the slightest sign of chest discomfort, extreme tiredness, sweating, and shortness of breath; SpO_2_ was measured to verify hypoxemia, and if a patient had SpO_2_ < 88%, the physical exercise was immediately terminated (Yang and Yang, 2020).

### 2.13 Nutritional intervention

The nutritional intervention was focused on the Food Guide for the Brazilian population (Brasil Ministério da Saude, 2014) to instruct participants about healthy eating, quality of life, and the importance of reducing risks associated with chronic non-communicable diseases (NCDs). Nutritional interventions were performed once a week in groups.

### 2.14 Psychoeducation

Psychoeducation was based on therapeutic interventions to provide knowledge and the possibility of change in the face of the psychological consequences of the COVID-19 pandemic based on a model of treatment and prevention of psychiatric illnesses (Authier, 1977). In this sense, they were asked about the importance of physical exercise, anxiety, factors associated with obesity, the role of food, stress, insomnia, fear, and binge eating (Ryal et al., 2023). Therapeutic interventions were performed once a week in groups.

### 2.15 Statistical analysis

All statistical analyses were performed using GraphPad Prism 8.1.0 software. Previously, the normality of the data was tested using the Shapiro–Wilk test. Similarly, the homogeneity of the data was tested by Levene’s test. After confirming normality and homogeneity, the numerical data were expressed as the mean and standard deviation (±SD), and the categorical data were expressed as absolute and relative frequency (%). Mauchly’s test of sphericity was used to test the Greenhouse‒Geisser correction, if necessary. To analyze the clinical characteristics, a one-way analysis of variance (ANOVA) was used to compare numerical data, and the chi-square non-parametric statistical test was used to compare categorical data. Two-way mixed-measures analysis of variance (ANOVA) was used to compare the groups and time (pre- and post-intervention). The Bonferroni *post hoc* test was used when a significant difference was found. A paired t-test (pre-*vs.* post-intervention) was applied when a time difference was detected to identify possible statistical significance in intra-groups conditions (Franchini et al., 2016), and the confidence interval (CI) was also calculated. The effect size was calculated using Cohen’s *d* (Cohen, 2013) as follows: 0.2 (*small effect*), 0.5 (*moderate effect*), and 0.8 (*large effect*). The effect size for eta-square (*ŋ* ^*2*^) was also calculated conforming proposed by Richardson (Richardson, 2011): 0.0099 (*small effect*), 0.0588 (*moderate effect*), 0.1379 (*large effect*). The significance level established was *p* ≤ 0.05.

## 3 Results

Table 2 presents the clinical characteristics of the present study participants stratified by the symptoms of COVID-19: severe (*n =* 7), moderate (*n =* 17), and the control group, without the diagnosis of COVID-19 (*n =* 11). No differences were observed for age (*p* = 0.09), BMI (*p* = 0.83), resting heart rate (*p* = 0.10), SBP (*p* = 0.32), DPB (*p* = 0.37), and SpO_2_ (*p* = 0.29). Regarding persistent symptoms self-reported by the COVID-19 participants (moderate and severe COVID-19 groups), fatigue (severe: 85.7%; moderate: 64.7%), memory deficit (severe: 85.7%; moderate: 45.1%) and difficult concentration (severe: 71.4%; moderate: 41.1%) were more prevalent. However, no significant difference between groups was observed for fatigue (*p* = 0.30), memory deficit (*p* = 0.08), and difficulty concentration (*p* = 0.18). In addition, differences were not detected for the other clinical characteristics (medical history, medication in use, self-reported post-COVID-19 symptoms, smoking, and physical activity: *p >* 0.05).

**TABLE 2:** Clinical characteristics of participants of the severe COVID-19*,* moderate COVID-19*,* and control groups.

Variables	Severe	Moderate	Control	*p-*value
Age (years old)	45 ± 10.0	51 ± 13.9	42 ± 9.1	*p* = 0.09
Gender				*p* = 0.30
Male	6 (85.7%)	11 (64.7%)	10 (90.9%)	
Female	1 (14.3%)	6 (35.3%)	1 (9.1%)	
BMI (kg/m^2^)	29.7 ± 2.3	32.5 ± 8.4	30.8 ± 7.9	*p* = 0.83
Medical history				
Hypertension	1 (14.3%)	3 (17.6%)	1 (9.1%)	*p* = 0.82
Diabetes	1 (14.3%)	1 (5.9%)	0 (0%)	*p* = 0.12
Dyslipidemia	0 (0%)	2 (11.8%)	0 (0%)	*p* = 0.34
COPD	0 (0%)	0 (0%)	0 (0%)	-
Asthma	0 (0%)	0 0%)	0 (0%)	-
CAD/revascularization	3 (42.8%)	2 (11.8%)	0 (0%)	*p* = 0.09
Others	1 (14.3%)	5 (29.4%)	0 (0%)	*p* = 0.13
Smoking				*p* = 0.30
No	2 (28.5%)	7 (41.1%)	7 (63.6%)	
Past or today	5 (71.4%)	10 (58.8%)	4 (36.3%)	
Medications in use				
Antihypertensive	3 (42.8%)	4 (23.5%)	1 (9.1%)	*p* = 0.22
Antidiabetic	0 (0%)	1 (5.9%)	0 (0%)	*p* = 0.58
Statin	0 (0%)	1 (5.9%)	0 (%)	*p* = 0.58
Platelet antiaggregant	0 (0%)	0 (0%)	0 (0%)	-
Anticoagulant	0 (0%)	1 (5.9%)	0 (0%)	*p* = 0.64
Others	0 (0%)	1 (5.9%)	1 (9.1%)	*p* = 0.14
Post-COVID-19 symptoms self-reported				
Fatigue	6 (85.7%)	11 (64.7%)	-	*p* = 0.30
Dyspnoea	3 (42.8%)	6 (35.3%)	-	*p* = 0.73
Muscle pain	4 (57.1%)	5 (29.4%)	-	*p* = 0.20
Joint pain	4 (57.1%)	5 (29.4%)	-	*p* = 0.20
Headache	1 (14.3%)	4 (23.5%)	-	*p* = 0.61
Dizziness	2 (28.5%)	4 (23.5%)	-	*p* = 0.80
Memory deficit	6 (85.7%)	8 (45.1%)	-	*p* = 0.08
Difficulty concentrating	5 (71.4%%)	7 (41.1%)	-	*p* = 0.18
Feeling of hearing loss	1 (14.3%)	3 (17.6%)	-	*p* = 0.84
Hair loss	4 (57.1%)	6 (35.3%)	-	*p* = 0.32
Loss of smell	1 (14.3%)	4 (23.5%)	-	*p* = 0.73
Physical activity ≥ 150 min/week	3 (42.8%)	6 (35.3%)	5 (45.4%)	*p* = 0.85
Baseline vital signs				
HR (bpm)	90 ± 15.8	82 ± 10.1	91 ± 10.3	*p* = 0.10
SBP (mmHg)	120 ± 10	120 ± 9.3	125 ± 7.5	*p* = 0.32
DBP (mmHg)	80 ± 8.1	80 ± 8.1	80 ± 4.9	*p* = 0.37
% SpO_2_	95 ± 0.9	96 ± 1.8	96 ± 0.9	*p* = 0.29

Note: numerical data are expressed as mean ± standard deviation, and categorical data are expressed as absolute and relative frequency (%); BMI, body mass index; COPD, chronic obstructive pulmonary disease; CAD, coronary artery disease; HR, heart rate; SBP, systolic blood pressure; DBP, diastolic blood pressure; %SpO_2_: oxygen saturation; significance level *p<* 0.05.

### 3.1 Body composition

**FIGURE 3:**
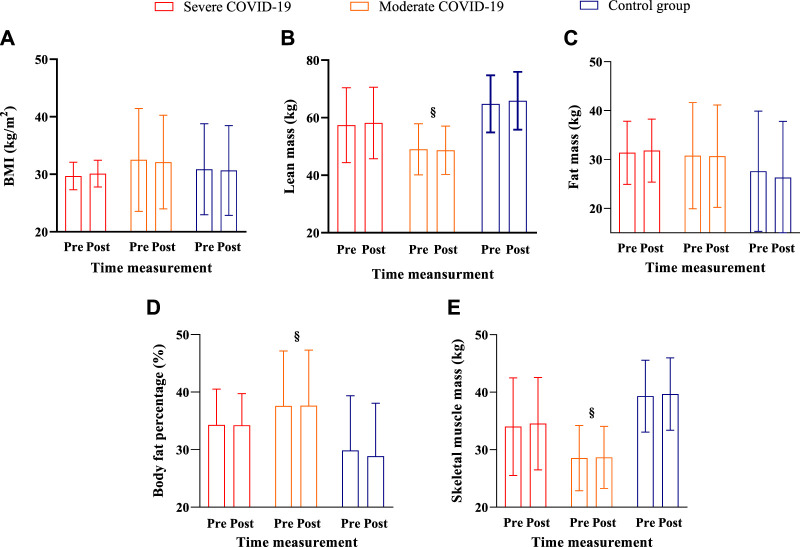
Body composition in the pre- and post-assessment interventions for the severe COVID-19, moderate COVID-19, and control groups. Note: Data are expressed as the mean ± standard deviation; § = a group difference with lower values for the moderate group compared to the control group; significance level established *p <* 0.05. **(A)** body mass; **(B)** lean mass; **(C)** fat mass; **(D)** body fat percentage (%), and **(E)** skeletal muscle mass.

Figure 3 shows the pre- and post-intervention body composition assessments.

For BMI, no group (F_2,28_ = 0.46; *p =* 0.63; *ŋ* ^*2*^ = 0.03; *small effect*), no time (F_1,28_ = 0.18; *p =* 0.66; *ŋ* ^*2*^ = 0.006; *small effect*) and no interaction effects (F_2,28_ = 2.85; *p =* 0.07; *ŋ* ^*2*^ = 0.16; *large effect*) were observed.

For lean mass, a group difference was detected (F_2,29_ = 8.02; *p =* 0.001; *ŋ* ^*2*^ = 0.35; *large effect*), with the Bonferroni *post hoc* test showing higher values for the control group when compared to the moderate group (*p =* 0.001). However, no time (F_1,29_ = 2.86; *p =* 0.10; *ŋ* ^*2*^ = 0.08; *medium effect*) and no interaction effects (F_2,29_ = 0.52; *p =* 0.59; *ŋ* ^*2*^ = 0.30; *large effect*) were detected.

For fat mass, no group (F_2,28_ = 0.50; *p =* 0.61; *ŋ* ^*2*^ = 0.03; *small effect*), no time (F_1,28_ = 2.28; *p =* 0.14; *ŋ* ^*2*^ = 0.07; *medium effect*), and no interaction effects (F_2.28_ = 2.33; *p =* 0.11; *ŋ* ^*2*^ = 0.14; *large effect*) were observed.

For body fat percentage, a group difference was detected (F_2,28_ = 3.89; *p =* 0.03; *ŋ* ^*2*^ = 0.21; *large effect*), with the Bonferroni *post hoc* test indicating lower values for the control group than the moderate group (*p =* 0.03). However, no time (F_1,28_ = 4.05; *p =* 0.05; *ŋ* ^*2*^ = 0.12; *large effect*) and no interaction effects (F_2,28_ = 0.95; *p =* 0.39; *ŋ* ^*2*^ = 0.06; *medium effect*) were found.

For skeletal muscle mass, a group difference (F_2,29_ = 8.52; *p =* 0.001; *ŋ* ^*2*^ = 0.37; *large effect*) was observed, with the Bonferroni *post hoc* test indicating higher values for the control when compared to the moderate COVID-19 group (*p =* 0.0008). However, no time (F_1,29_ = 3.76; *p =* 0.06; *ŋ* ^*2*^ = 0.11; *medium effect*) and no interaction effects (F_2,29_ = 0.04; *p =* 0.95; *ŋ* ^*2*^ = 0.003; *small effect*) were detected.

### 3.2 Health-related physical fitness tests

**FIGURE 4:**
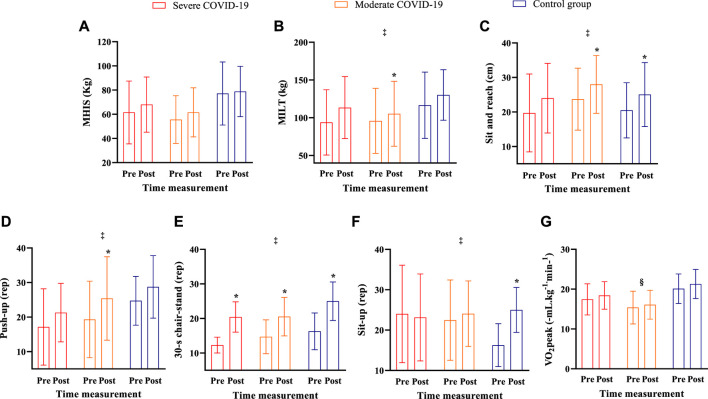
Physical tests in the pre- and post-assessment interventions for the severe COVID-19, moderate COVID-19*,* and control groups. Note: Data are expressed as the mean ± standard deviation; § = a group difference with lower values for the moderate group when compared to the control group; **‡ =** time difference from post-intervention; * = significant difference between pre- and post-intervention for the same group intervention; significance level established *p <* 0.05. **(A)** MIHS; **(B)** MILT; **(C)** sit and reach; **(D)** push-up; **(E)** 30-s chair-stand; **(F)** sit-up and **(G)** VO_2_peak.

Figure 4 shows the physical fitness tests of the COVID-19 groups and a control group before and after 8 weeks of intervention.

For sit and reach test, no group (F_2,31_ = 0.66; *p =* 0.52; *ŋ* ^*2*^ = 0.04; *small effect*) and no interaction effects were observed (F_2,31_ = 0.01; *p =* 0.98; *ŋ* ^*2*^ = 0.001; *small effect*). However, a time difference was detected (F_1,31_ = 32.32; *p =* 0.000003; *ŋ* ^*2*^ = 0.51; *large effect*), with the Bonferroni *post hoc* test indicating a significant increase after 8 weeks of intervention (*p =* 0.000001). When each group was analyzed and isolated by the t-test, this difference was not confirmed for the severe COVID-19 group (*t*_7_ = 2.034; *p =* 0.08; CI: −0.8695 to 9.441; *d = 0.40; moderate effect*). However, isolated t-test revealed higher values in post than pre-intervention in moderate COVID-19 (*t*_16_ = 4.308; *p =* 0.0006; CI: 2.163 to 6.399; *d =0.04; small effect*) and control groups (*t*_11_ = 4.125; *p =* 0.0021; CI: 2.098 to 7.029; *d = 0.05; small effect*).

For MIHS, no group (F_2,32_ = 2.87; *p =* 0.07; *ŋ* ^*2*^ = 0.15; *large effect*), no time (F_2,32_ = 3.75; *p =* 0.06; *ŋ* ^*2*^ = 0.10; *large effect*) and no interaction effects (F_2,32_ = 0.41; *p =* 0.66; *ŋ* ^*2*^ = 0.02; *small effect*) were observed.

For MILT, no group (F_2,32_ = 1.12; *p =* 0.33; *ŋ* ^*2*^ = 0.06; *medium effect*) and no interaction effects were detected (F_2,32_ = 0.64; *p =* 0.52; *ŋ* ^*2*^ = 0.03; *large effect*). However, a time effect was found (F_1,32_ = 15.35; *p =* 0.0004; *ŋ* ^*2*^ = 0.32; *large effect*), with the Bonferroni *post hoc* test indicating a significant increase after 8 weeks of intervention (*p =* 0.0006). When each group was analyzed and isolated by the t-test, this difference was not confirmed for the severe COVID-19 (*t*_7_ = 1.566; *p =* 0.168; CI: −3.658 to 16.6; *d =0.26; small effect*) and control groups (*t*_11_ = 0.3145; *p =* 0.75; CI: −10.07 to 13.38; *d =0.07; small effect*)*.* However, the isolated t-test revealed higher values in post than pre-intervention in the moderate COVID-19 group (*t*_17_ = 2.178; *p =* 0.04; CI: 0.1601 to 11.79; *d = 0.29; small effect*)*.*

For push-up test, no group (F_2,30_ = 1.37; *p =* 0.26; *ŋ* ^*2*^ = 0.08; *medium effect*) and no interaction effects were detected (F_2,30_ = 0.12; *p =* 0.88; *ŋ* ^*2*^ = 0.008; *small effect*). However, a time effect was observed (F_1,30_ = 12.23; *p =* 0.001; *ŋ* ^*2*^ = 0.28; *large effect*), with the Bonferroni *post hoc* test indicating a significant increase in push-up test after 8 weeks of intervention (*p =* 0.0006). When each group was analyzed and isolated by the t-test, this difference was not confirmed for the severe COVID-19 (*t*_7_ = 1.442; *p =* 0.19; CI: −2.889 to 11.17; *d = 0.06; small effect*) and control groups (*t*_11_ = 1.888; *p =* 0.08; CI: −0.7218 to 8.722; *d =0.04; small effect*)*.* Nonetheless, the isolated t-test indicated higher values in post than pre-intervention in the moderate COVID-19 group (*t*_15_ = 3.048; *p =* 0.008; CI: 1.561 to 8.972; *d = 0.52; moderate effect*)*.*

For the 30-s chair-stand test, no group (F_2,32_ = 3.03; *p =* 0.05; *ŋ* ^*2*^ = 0.16; *large effect*) and no interaction effects were observed (F_2,32_ = 0.80; *p =* 0.45; *ŋ* ^*2*^ = 0.04; *small effect*). A time effect was observed (F_1,32_ = 43.95; *p =* 0.0000000; *ŋ* ^*2*^ = 0.57; *large effect*) with a significant increase in repetitions confirmed by Bonferroni *post hoc* test (*p =* 0.000000). The isolated t-test for each group indicated an increase in the repetitions performed in the severe COVID-19 (*t*_7_ = 5.362; *p =* 0.001; CI: 4.427 to 11.86; *d =0.23; small effect*), moderate COVID-19 (*t*_17_ = 3.460; *p =* 0.003; CI: 2.256 to 9.391; *d = 0.11; small effect*) and control groups (*t*_11_ = 4.498; *p =* 0.001; CI: 4.404 to 13.05; *d = 0.16; small effect*)*.*

For the sit-up test, no group (F_2,26_ = 0.62; *p =* 0.54; *ŋ* ^*2*^ = 0.04; *small effect*) and no interaction effects were observed (F_2,26_ = 3.24; *p =* 0.05; *ŋ* ^*2*^ = 0.19; *large effect*). However, a time difference was verified (F_1,26_ = 5.46; *p =* 0.02; *ŋ* ^*2*^ = 0.17; *large effect*), with the Bonferroni *post hoc* test indicating higher values after 8 weeks of intervention (*p =* 0.004). Isolated t-test did not confirm this effect for the severe COVID-19 (*t*_5_ = 0.1721; *p =* 0.87; CI: −10.28 to 9.082; *d = 0.07; small effect*) and moderate COVID-19 groups (*t*_13_ = 1.130; *p =* 0.28; CI: −2.426 to 7.656; *d = 0.01; small effect*)*.* However, the isolated t-test indicated increased repetitions performed in the control group (*t*_11_ = 4.498; *p =* 0.0011; CI: 4.404 to 13.05; *d = 0.16; small effect*)*.*

For VO_2_ peak, a group difference was found (F_2,31_ = 5.96; *p =* 0.006; *ŋ* ^*2*^ = 0.27; *large effect*), with the Bonferroni *post hoc* test indicating higher values for the control group when compared to moderate COVID-19 group (*p =* 0.004). However, no time (F_1,31_ = 3.96; *p =* 0.05; *ŋ* ^*2*^ = 0.11; *medium effect*) and no interaction effects (F_2,31_ = 0.29; *p =* 0.74; *ŋ* ^*2*^ = 0.01; *small effect*) were observed for cardiorespiratory fitness.

### 3.3 Training monitoring

Table 3 presents the training monitoring results of the three experimental groups (moderate and severe COVID-19 and a control group) before and after 8 weeks of intervention.

**TABLE 3:** Training monitoring of the severe COVID-19*,* moderate COVID-19*,* and control groups in pre- and post-assessment intervention.

Variables	Severe		Moderate		Control	
	Pre	Post	Pre	Post	Pre	Post
% SpO_2_ initial^a^	96 ± 1	97 ± 2	96 ± 2	97 ± 2	97 ± 2	97 ± 1
% SpO_2_ final	93 ± 3	93 ± 5	95 ± 3	96 ± 1	96 ± 3	95 ± 2
SBP initial (mmHg)	120 ± 7	120 ± 7	120 ± 15	120 ± 8	120 ± 10	130 ± 9
SBP final (mmHg)^b^	130 ± 13	120 ± 10^c^	130 ± 13	120 ± 13	130 ± 13	130 ± 13
DBP initial (mmHg)	80 ± 8	70 ± 12	80 ± 8	70 ± 9	80 ± 9	70 ± 12
DBP final (mmHg)^b^	80 ± 9	70 ± 10	80 ± 7	70 ± 8^c^	80 ± 7	70 ± 9
RPE (*u.a*) ^b^	5 ± 1	7 ± 1	6 ± 1	6 ± 2	5 ± 1	7 ± 1^c^
RPR (*u.a*)^b^	7 ± 2	9 ± 1	7 ± 2	8 ± 1	8 ± 1	8 ± 1
Tonnage (kg) ^b^	3,507 ± 2,615	11,156 ± 5545^c^	3,967 ± 2,123	8,713 ± 4346^c^	6,005 ± 1855	11,602 ± 8,690

Note: data expressed as mean and ±standard deviation; %SpO_2_ = peripherical oxygen saturation; SBP, systolic blood pressure; DBP, diastolic blood pressure; RPE, rating perceived exertion; RPR, rating perceived recovery.

^a^ a group difference with lower values for the severe and moderate group when compared to the control group.

^**b**^ time difference from post-intervention.

^c^ significant difference between pre- and post-intervention for the same group intervention; significance level established *p*< 0.05.

For initial SpO_2,_ a group difference (F_2,31_ = 5.3; *p =* 0.01; *ŋ* ^*2*^ = 0.25; *large effect*) was detected with higher values for the control group when compared to moderate COVID-19 (*p =* 0.04) and severe COVID-19 groups (*p =* 0.01). A time difference was detected (F_1,31_ = 5.0; *p =* 0.03; *ŋ* ^*2*^ = 0.13; *large effect*), but the Bonferroni *post hoc* did not confirm these findings (*p* > 0.05). Besides that, no interaction effect was observed (F_2,31_ = 0.8; *p =* 0.46; *ŋ* ^*2*^ = 0.04; *small effect*).

About the final SpO_2_, a group difference was observed (F_2,32_ = 3.32; *p =* 0.04; *ŋ* ^*2*^ = 0.17; *large effect*), but the Bonferroni *post hoc* did not confirm these differences (*p* > 0.05). Besides that, no time (F_1,32_ = 0.85; *p =* 0.36; *ŋ* ^*2*^ = 0.02; *small effect*), and no interaction effects (F_2,32_ = 1.02; *p =* 0.37; *ŋ* ^*2*^ = 0.05; *small effect*) were found.

For initial SBP, no group (F_2,31_ = 0.04; *p =* 0.95; *ŋ* ^*2*^ = 0.002; *small effect*), no time (F_1,31_ = 0.02; *p =* 0.88; *ŋ* ^*2*^ = 0.00007; *small effect*) and no interaction effects (F_2,31_ = 1.22; *p =* 0.30; *ŋ* ^*2*^ = 0.07; *medium effect*) were detected.

For the final SBP, no group (F_2,30_ = 0.01; *p =* 0.98; *ŋ* ^*2*^ = 0.0008; *small effect*) and no interaction effects were detected (F_2,30_ = 0.29; *p =* 0.74; *ŋ* ^*2*^ = 0.01; *small effect*). A time effect was observed (F_1,30_ = 10.61; *p =* 0.002; *ŋ* ^*2*^ = 0.26; *large effect*), with the Bonferroni *post hoc* test indicating lower values after 8 weeks of intervention (*p =* 0.003). An isolated t-test revealed lower values in post than pre-intervention in the severe COVID-19 group (*t*_7_ = 2.500; *p =* 0.04; CI: −28.273 to 0.3034; *d = 0.12; large effect*)*.* However, when each group was analyzed and isolated by the t-test, this difference was not confirmed for the moderate COVID-19 (*t*_15_ = 1.702; *p =* 0.11; CI: −19.13 to 2.200; *d = 0.05; small effect*) and control groups (*t*_11_ = 1.742; *p =* 0.11; CI: −19.68 to 2.412; *d = 0.05; small effect*)*.*

For initial DBP, no group (F_2,31_ = 0.22; *p =* 0.80; *ŋ* ^*2*^ = 0.01; *small effect*), no time (F_1,31_ = 3.46; *p =* 0.07; *ŋ* ^*2*^ = 0.10; *large effect*) and no interaction effects (F_2,31_ = 0.16; *p =* 0.84; *ŋ* ^*2*^ = 0.01; *small effect*) were observed.

For the final DBP, no group (F_2,30_ = 2.41; *p =* 0.10; *ŋ* ^*2*^ = 0.13; *large effect*) and no interaction effects were observed (F_2,30_ = 0.29; *p =* 0.74; *ŋ* ^*2*^ = 0.01; *small effect*). A time difference was detected (F_1,30_ = 13.32; *p =* 0.0009; *ŋ* ^*2*^ = 0.30; *large effect*) with the Bonferroni *post hoc* test indicating lower values after 8 weeks of intervention (*p =* 0.0004). When each group was analyzed and isolated by the t-test, this difference was not confirmed for the severe COVID-19 (*t*_7_ = 1.698; *p =* 0.14; CI: −17.43 to 3.148; *d = 0.10; small effect*) and control groups (*t*_11_ = 1.604; *p =* 0.13; CI: −13.03 to 2.124; *d = 0.06; small effect*)*.* However, the isolated t-test revealed lower values in post than pre-intervention in moderate COVID-19 group (*t*_15_ = 3.389; *p =* 0.004; CI: −14.15 to −3.182; *d = 0.11; small effect*)*.*

For the RPE, no group (F_2,23_ = 0.22; *p =* 0.79; *ŋ* ^*2*^ = 0.01; *small effect*) and no interaction effects were detected (F_2,23_ = 2.67; *p =* 0.09; *ŋ* ^*2*^ = 0.18; *large effect*). A time difference was found (F_1,23_ = 12.57; *p =* 0.001; *ŋ* ^*2*^ = 0.35; *large effect*), with the Bonferroni *post hoc* test indicating higher values after 8 weeks of intervention (*p =* 0.0038). When each group was analyzed and isolated by the t-test, this difference was not confirmed for the severe COVID-19 (*t*_5_ = 1.800; *p =* 0.10; CI: −0.5884 to 4.188; *d =1.48; large effect*) and moderate COVID-19 groups (*t*_12_ = 0.5567; *p =* 0.58; CI: −0.8614 to 1.445; *d = 0.17; small effect*). However, the isolated t-test revealed higher values in post than pre-intervention in the control group (*t*_9_ = 1.778; *p =* 0.01; CI: 0.4588 to 3.097; *d = 0.24; small effect*).

For the RPR, no group (F_2,23_ = 0.63; *p =* 0.53; *ŋ* ^*2*^ = 0.05; *small effect*) and no interaction effects were observed (F_2,23_ = 1.26; *p =* 0.30; *ŋ* ^*2*^ = 0.09; *medium effect*). Nonetheless, a time difference was observed for the RPR (F_1,23_ = 9.59; *p =* 0.005; *ŋ* ^*2*^ = 0.29; *large effect*), and the Bonferroni *post hoc* test indicating higher values after 8 weeks of intervention (*p =* 0.01). When each group was analyzed isolated by the t-test, this difference was not confirmed for the severe COVID-19 (*t*_5_ = 1.871; *p =* 0.13; CI: −1.017 to 5.217; *d = 0.26; small effect*), moderate COVID-19 (*t*_12_ = 1.689; *p =* 0.11; CI: −0.3160 to 2.399; *d =0.63; moderate effect*) and control groups (*t*_9_ = 1.189; *p =* 0.26; CI: −0.5220 to 1.633; *d = 0.36; small effect*).

### 3.4 Tonnage

Table 3 presents the tonnage results before and after 8 weeks of intervention.

For tonnage, no group (F_2,26_ = 1.15; *p =* 0.33; *ŋ* ^*2*^ = 0.08; *medium effect*) and no interaction effects were observed (F_2,26_ = 0.53; *p =* 0.59; *ŋ* ^*2*^ = 0.03; *small effect*). A time effect was detected for tonnage after intervention (F_1,26_ = 22.03; *p =* 0.00007; *ŋ* ^*2*^ = 0.45; *large effect*), with the Bonferroni *post hoc* showing higher values after intervention (*p =* 0.00008). When each group was analyzed isolated by the t-test, this difference was confirmed for the severe COVID-19 (*t*_6_ = 3.576; *p =* 0.01; CI: 2,151 to 13,148; *d = 1.76; large effect;* Δ = 218.1%) and moderate COVID-19 groups (*t*_14_ = 4.827; *p =* 0.0003; CI: 2,705 to 7,089; *d =1.38; large effect;* Δ = 119.3%)*,* but this effect was not confirmed for the control group (*t*_9_ = 1.687; *p =* 0.13; CI: −2,122 to 13,680; *d = 0.89; large effect;* Δ = 93.1%)*.* However, 8 weeks of intervention increased the tonnage of the experimental groups (severe: 218.1%; moderate: 119.3%; control: 93.1%) compared to the pre-intervention time.

### 3.5 Biochemical parameters

The analyses of the biomarkers, i.e., fasting glucose, HbA1C, lipid profile, liver enzymes, CRP, and delta percentage values, are presented in Table 4.

**TABLE 4:** Biochemical parameters of the severe COVID-19, moderate COVID-19, and control group in pre- and post-assessment intervention.

Variables	Severe				Moderate				Control		
	Pre	Post	Δ%		Pre	Post	Δ%		Pre	Post	Δ%
Fasting glucose (mg/dL)^a^	101.4 ± 15.8	96.1 ± 11.7	−4.5		95.9 ± 9.4	92.0 ± 13.3^b^	−6.2		96.9 ± 8.0	88.9 ± 8.6^b^	−8.3
HbA1C (%)	6.5 ± 0.6	6.1 ± 0.3	−5.9		6.1 ± 0.5	6.1 ± 0.8	−3.6		6.5 ± 0.7	6.1 ± 0.7	−6.3
Total cholesterol (mg/dL)^a^	175.5 ± 22.4	166.0 ± 23.1	−5.5		177.7 ± 33.2	157.9 ± 33.9	1.1		200.1 ± 44.9	177.1 ± 16.3^b^	−8.9
HDL-c (mg/dL)^a^	47.2 ± 6.8	52.1 ± 5.9^b^	11.2		54.6 ± 15.1	57.3 ± 12.9	6.2		52.7 ± 14.8	54.0 ± 12.2	3.1
LDL-c (mg/dL)	97.3 ± 23.6	91.3 ± 26.0	−7.2		93.1 ± 25.2	91.1 ± 27.1	−0.8		103.7 ± 26.5	99.1 ± 32.1	0.7
TGL (mg/dL)^a^	155.1 ± 59.3	112.2 ± 51.8^b^	−28.2		149.5 ± 79.6	91.0 ± 35.1^b^	−15.6		179.6 ± 101.6	147.7 ± 74.0^b^	−31.0
ALT (U/L)	35.8 ± 12.8	28.0 ± 7.6	−19.5		29.0 ± 16.0	29.8 ± 14.2	12.0		37.9 ± 18.9	40.3 ± 19.1	38.5
AST (U/L)	30.7 ± 8.9	27.4 ± 7.2	−6.7		33.0 ± 15.6	29.8 ± 5.8	−4.3		33.6 ± 12.1	38.1 ± 17.7	22.8
ALP (U/L)	63.0 ± 16.7	60.4 ± 20.3	−2.9		67.5 ± 15.1	72.1 ± 16.9	5.6		66.3 ± 12.6	67.4 ± 13.2	4.0
GAMMA-GT (U/L)	44.5 ± 12.6	39.7 ± 9.5	−9.8		38.2 ± 18.3	38.5 ± 17.3	11.7		38.2 ± 16.4	55.1 ± 27.2	45.1
Albumin (g/dL)^a^	4.1 ± 0.2	4.6 ± 0.2^b^	11.9		4.0 ± 0.3^b^	4.5 ± 0.1	12.2		4.3 ± 0.3	4.5 ± 0.1	6.1
CRP (mg/L)^a^	10.1 ± 8.9	2.5 ± 1.7	−56.6		8.9 ± 6.2	3.1 ± 3.2^b^	−68.4		11.7 ± 4.8	4.0 ± 4.5^b^	−25.1

Note: data expressed as mean and ±standard deviation; Δ% = relative delta; HDL-c, high-density lipoprotein; LDL-c, low-density lipoprotein; TGL, triglycerides; ALT, alanine aminotransferase; AST, aspartate aminotransferase; ALP, alkaline phosphatase; GAMMA-GT, Gamma-glutamyl transferase; CRP = C-reactive protein; Δ = relative delta.

^a^ time difference from post-intervention.

^b^ significant difference between pre− and post-intervention for the same group intervention; significance level established *p*<0.05.

For CRP, no group (F_2,28_ = 0.56; *p =* 0.57; *ŋ* ^*2*^ = 0.03; *small effect*) and no interaction effects were observed (F_2,28_ = 0.15; *p =* 0.86; *ŋ* ^*2*^ = 0.01; *small effect*). However, a time difference was detected (F_1,28_ = 26.53; *p =* 0.000018; *ŋ* ^*2*^ = 0.48; *large effect*), with the Bonferroni *post hoc* test indicating a significant reduction in CRP after 8 weeks of intervention (*p =* 0.0001). The isolated t-test confirm this difference for the severe COVID-19 (*t*_7_ = 2.346; *p =* 0.05; CI: −15.52 to 0.3268; *d = 0.23; small effect*)*,* moderate COVID-19 (*t*_13_ = 3.582; *p =* 0.003; CI: −9.858 to −2.402; *d =1.17; large effect*), and control groups (*t*_11_ = 3.148; *p =* 0.01; CI: −13.02 to −2.227; *d = 0.61; moderate effect*).

For albumin, no group (F_2,28_ = 2.07; *p =* 0.14; *ŋ* ^*2*^ = 0.12; *large effect*) and no interaction effects were verified (F_2,28_ = 3.03; *p =* 0.06; *ŋ* ^*2*^ = 0.17; *large effect*). However, a time effect was detected (F_1,28_ = 39.0; *p =* 0.000001; *ŋ* ^*2*^ = 0.58; *large effect*), with the Bonferroni *post hoc* test showing higher values in post-intervention (*p =* 0.000001). The isolated t-test for each group indicated an increase in the values for the severe COVID-19 (*t*_7_ = 5.160; *p =* 0.002; CI: 0.2584 to 0.7244; *d =0.23; small effect*) and moderate COVID-19 groups (*t*_13_ = 4.730; *p =* 0.0005; CI: 0.2381 to 0.6449; *d = 0.17; small effect*)*.* However, *the* isolated t-test did not confirm this difference for the control group (*t*_11_ = 1.712; *p =* 0.11; CI: −0.05070 to 0.3871; *d = 0.06; small effect*)*.*

For fasting glucose, no group (F_2,27_ = 1.08; *p =* 0.35; *ŋ* ^*2*^ = 0.07; *medium effect*) and no interaction effects were observed (F_2,27_ = 0.34; *p =* 0.71; *ŋ* ^*2*^ = 0.02; *small effect*). A time effect was detected (F_1,27_ = 26.58; *p =* 0.00002; *ŋ* ^*2*^ = 0.49; *large effect*), with the Bonferroni *post hoc* test indicating a significant reduction after 8 weeks of intervention (*p =* 0.000009). When each group was analyzed and isolated by the t-test, this difference was not confirmed for the severe COVID-19 group (*t*_7_ = 1.409; *p =* 0.20; CI: −14.47 to 3.896; *d = 6.15; large effect*)*.* However, isolated t-test revealed lower values in post than pre-intervention in moderate COVID-19 (*t*_12_ = 4.084; *p =* 0.001; CI: −10.13 to −3.036; *d = 0.05; small effect*) and control groups (*t*_11_ = 4.619; *p =* 0.001; CI: −11.86 to −4.141; *d =0.09; small effect*)*.*

For HbA1C, no group (F_2,27_ = 1.65; *p =* 0.20; *ŋ* ^*2*^ = 0.10; *medium effect*), no time (F_1.27_ = 3.73; *p =* 0.06; *ŋ* ^*2*^ = 0.12; *medium effect*), and no interaction (F_2,27_ = 0.29; *p =* 0.75; *ŋ* ^*2*^ = 0.21; *large effect*) were found.

For total cholesterol, no group (F_2,26_ = 0.51; *p =* 0.60; *ŋ* ^*2*^ = 0.03; *small effect*) and no interaction effects were observed (F_2,26_ = 1.04; *p =* 0.36; *ŋ* ^*2*^ = 0.07; *medium effect*). A time difference was detected (F_1,26_ = 10.35; *p =* 0.003; *ŋ* ^*2*^ = 0.28; *large effect*), with the Bonferroni *post hoc* test indicating a significant reduction after 8 weeks of intervention (*p =* 0.0002). When each group was analyzed and isolated by the t-test, this difference was not confirmed for the severe COVID-19 (*t*_7_ = 2.359; *p =* 0.06; CI: −19.50 to 0.3587; *d = 0.04; small effect*) and moderate COVID-19 groups (*t*_12_ = 1.580; *p =* 0.14; CI: −21.74 to 3.573; *d =0.05; small effect*)*.* However, the isolated t-test revealed lower values in post than pre-intervention in the control group (*t*_10_ = 2.379; *p =* 0.04; CI: −42.14 to −1.059; *d =0.08; small effect*).

For HDL-c, no group (F_2,27_ = 0.93; *p =* 0.40; *ŋ* ^*2*^ = 0.06; *medium effect*) and no interaction effects were detected (F_2,27_ = 0.95; *p =* 0.39; *ŋ* ^*2*^ = 0.06; *medium effect*). A time effect was verified (F_1,27_ = 8.21; *p =* 0.007; *ŋ* ^*2*^ = 0.23; *large effect*), with the Bonferroni *post hoc* test indicating higher values after intervention (*p =* 0.01). When each group was analyzed and isolated by the t-test, this difference was not confirmed for the moderate COVID-19 (*t*_13_ = 1.718; *p =* 0.11; CI: −0.6721 to 5.688; *d = 0.19; small effect*) and control groups (*t*_10_ = 0.6141; *p =* 0.55; CI: −3.462 to 6.042; *d = 0.09; small effect*). However, isolated t-test revealed higher values in post than pre-intervention in the severe COVID-19 group (*t*_7_ = 4.186; *p =* 0.005; CI: 2.060 to 7.857; *d = 0.07; small effect*).

For LDL-c, no group (F_2,28_ = 0.22; *p =* 0.79; *ŋ* ^*2*^ = 0.01; *small effect*), no time (F_1,28_ = 1.58; *p =* 0.21; *ŋ* ^*2*^ = 0.05; *small effect*) and no interaction effects (F_2,28_ = 0.29; *p =* 0.74; *ŋ* ^*2*^ = 0.02; *small effect*) were observed.

For TGL, no group difference (F_2,27_ = 1.30; *p =* 0.28; *ŋ* ^*2*^ = 0.08; *medium effect*) and no interaction effects were observed (F_2,27_ = 0.02; *p =* 0.97; *ŋ* ^*2*^ = 0.001; *small effect*). Nonetheless, a time difference was found (F_1,27_ = 19.49; *p =* 0.0001; *ŋ* ^*2*^ = 0.41; *large effect*), with the Bonferroni *post hoc* test indicating a significant decrease after intervention (*p =* 0.00008). Isolated t-test revealed lower values in post than pre-intervention in severe COVID-19 (*t*_7_ = 4.597; *p =* 0.003; CI: −65.67 to −20.04; *d = 0.07; small effect*)*,* moderate COVID-19 (*t*_13_ = 2.789; *p =* 0.01; CI: −86.87 to −10.67; *d = 1.23; large effect*) and control groups (*t*_10_ = 2.491; *p =* 0.03; CI: −87.96 to −4.236; *d = 0.06; small effect*).

For ALT, no group (F_2,27_ = 1.05; *p =* 0.36; *ŋ* ^*2*^ = 0.07; *medium effect*), no time (F_1,27_ = 0.68; *p =* 0.41; *ŋ* ^*2*^ = 0.02; *small effect*) and no interaction effects (F_2,27_ = 2.54; *p =* 0.09; *ŋ* ^*2*^ = 0.15; *large effect*) were detected. For AST, no group (F_2,28_ = 1.12; *p =* 0.34; *ŋ* ^*2*^ = 0.07; *medium effect*), no time (F_1,28_ = 0.03; *p =* 0.84; *ŋ* ^*2*^ = 0.001; *small effect*) and no interaction effects (F_2,28_ = 0.85; *p =* 0.43; *ŋ* ^*2*^ = 0.05; *medium effect*) were observed. For ALP, no group (F_2,28_ = 1.11; *p =* 0.34; *ŋ* ^*2*^ = 0.07; *medium effect*) no time (F_1,28_ = 0.46; *p =* 0.49; *ŋ* ^*2*^ = 0.16; *large effect*), and no interaction effects (F_2,28_ = 1.18; *p =* 0.32; *ŋ* ^*2*^ = 0.07; *medium effect*) were detected. Finally, for GAMMA-GT, no group (F_2,27_ = 0.96; *p =* 0.39; *ŋ* ^*2*^ = 0.06; *medium effect*), no time (F_1,27_ = 1.89; *p =* 0.18; *ŋ* ^*2*^ = 0.06; *medium effect*), and no interaction effects (F_2,27_ = 2.77; *p =* 0.07; *ŋ* ^*2*^ = 0.17; *large effect*) were detected.

## 4 Discussion

The present study aimed to investigate the effects of multiprofessional intervention on body composition, physical fitness, and biomakers in overweight COVID-19 survivors. In summary, the main findings observed after 8 weeks of intervention were as follows: i) 8 weeks of multi-professional intervention did not produce significant improvements in body composition in the severe, moderate, and control COVID-19 groups; ii) no differences were observed for MIHS and VO_2_peak for all intervention groups; iii) the moderate COVID-19 group showed improvement in MILT, sit and reach, and push-up tests and the control group showed improvement in sit-up test; all intervention groups showed improvement in 30-s chair-stand test; iv) final SBP showed a significant reduction for the severe COVID-19 group, and DBP showed a significant reduction for moderate COVID-19 group; v) tonnage was higher in the last training session for moderate and severe COVID-19 groups; vi) CRP presented a significant reduction in moderate and control groups vii) albumin showed a significant improvement in moderate and severe COVID-19 groups; (viii) fasting glucose showed a significant reduction in moderate and control groups ix) total cholesterol showed a significant reduction in control group; x) HDL-c showed a significant improvement in severe COVID-19 group and ***xi*** *)* TG was reduced in all intervention groups. Consequently, the study’s hypothesis was not confirmed.

Despite not reducing the risk of infection by COVID-19, reducing body weight seems to be a protective measure against the worsening of COVID-19 disease, as it reduces the inflammatory processes caused by obesity (Queiroz et al., 2022). A previous study reported that hospitalized patients with COVID-19 showed higher values of fat mass and body fat percentage when compared to individuals who manifested the mild form with the same BMI (Lemos et al., 2022). Given this and supporting this perspective, regular physical exercise and healthy nutrition can help control these parameters and favor a better immune response against COVID-19 infection (Queiroz et al., 2022), regardless of disease symptomatology. Nonetheless, no significant BMI reductions or body composition improvements were observed at the end of the 8 weeks. The effects of the physical exercise program on body composition are directly related to the exercise dose (duration, intensity, and frequency), generating a negative energy balance and decreasing body fat (Gleeson et al., 2011). However, the lack of weight-loss success with physical exercise can be explained by compensatory responses that neutralize energy balance to maintain homeostasis (Flack et al., 2020). According to *Flack et al.* (Flack et al., 2020), individuals compensate for approximately 50% of the calories spent with physical exercise regardless of the exercise dose. In our study, we did not collect dietary records before and after the multi-professional interventions. Thus, we cannot establish a relationship between the participant’s body composition and dietary intake.

In addition, given the qualitative analyses carried out by our multi-professional team, there was a significant limitation of COVID-19 survivors who had moderate or severe disease cases regarding motor coordination to perform strength exercises. The moderate COVID-19 group improved MILT, sit and reach, push-ups, and 30-s chair-stand test, and the severe COVID-19 group just showed an improvement in the 30-s chair-stand test. These responses suggest that 8 weeks of multi-professional intervention could not be enough to promote progress in physical fitness in the severe COVID-19 group. Even though there is no previous evidence regarding the optimal tonnage for patients post-COVID-19 disease (Goldbaum et al., 2021), there was an improvement for moderate and severe COVID-19 groups. The severe COVID-19 group had an increase of 218.1%, and the moderate COVID-19 group increased by 119.3%, despite the physical limitations of the hospitalized patients.

When the present study was developed, there was no scientific basis for physical exercise prescription for individuals affected by COVID-19, specifically regarding the dosage (duration, intensity, and frequency of exercise) since the clinical condition of patients can be very heterogeneous. In the present study, the exercise sessions lasted approximately 60 min twice a week and were periodized to achieve a moderate intensity score via the RPE scale. Thus, our results indicated moderate classification by the experimental groups. We consider moderate-intensity exercise to be more appropriate due to the suppressive response of the immune system to high-intensity exercise. It is well documented that high-intensity physical exercise can decrease the immune system’s defense mechanisms, making the body sensitive to infection and viral reactivation for 3–72 h after the exercise session (Arazi et al., 2021). Therefore, prioritizing patient safety, the recommendations were followed to avoid strenuous and long-duration sessions in individuals more susceptible to viral infections. However, it is worth mentioning that the WHO later released guidelines for post-COVID-19 rehabilitation recommending 20–30 min of conditioning exercise five times a week and 3 sets of 10 repetitions of muscle-strengthening exercises three times a week but without specific specifications for control/reduction of body weight and positive changes in body composition (World Health Organization et al., 2021b).

Regarding health-related physical fitness tests, significant improvements were observed in the 30-s chair-stand test for the severe and moderate COVID-19 groups and control group after 8 weeks of intervention, independent of changes in anthropometric and body composition differences, corroborating the findings of *Li et al.* (Li et al., 2021) and *Dalbosco-Salas et al.* (Dalbosco-Salas et al., 2021) after 6 and 9 weeks of tele-exercises, respectively, posthospital discharge for COVID-19. The lack of significant differences in anthropometric and body composition was explained by a lockdown, in which people could not move freely and, consequently, spent low energy (Silva et al., 2022). It is worth mentioning that the participants needed sufficient intra- and inter-coordination to perform the exercises correctly and overcome inflammation (measured via CRP). No significant improvements were detected in sit and reach, push-up, sit-up, MILT, and MIHS for severe COVID-19. These findings do not corroborate those of *Everaerts et al.* (Everaerts et al., 2021), who found improved handgrip strength after 12 weeks of intervention in post-discharge patients.

Cardiorespiratory fitness was assessed using the VO_2_peak to verify physical capacity, effort tolerance, and possible cardiopulmonary abnormalities (Cahalin et al., 1996; ATS Committee on Proficiency Standards for Clinical Pulmonary Function Laboratories, 2002). The VO_2_peak of the control group was higher ​​than that of the moderate and severe COVID-19 groups. Furthermore, no significant difference was detected in VO_2_peak for all groups after the intervention, indicating possible lower stimulus to improve aerobic capacity. Contrary to the results of the present study, a previous study has shown improvements in VO_2_peak (Rinaldo et al., 2021) in response to concurrent training in patients after hospital discharge for COVID-19, which can be explained by differences in manipulations of training variables (duration, intensity, and frequency). The exercise dose can explain the absence of significant effects on cardiorespiratory fitness. According to *Bull et al.* (Bull et al., 2020), 150–300 min per week of moderate-intensity or 75–150 min of vigorous-intensity physical activity is the minimum necessary to maintain health status in eutrophic individuals, which did not occur in the present study due to the inability of the participants to visit the intervention location at a high enough frequency to achieve 120 min a week.

All physical exercises aimed at rehabilitation must be performed safely. Thus, SpO_2_ was monitored. The present study showed higher SpO_2_ values for the control group compared to both COVID-19 groups, but these responses could be expected according to the impacts of this disease (Lemos et al., 2022).

The final SBP was significative reduced in the final intervention for the severe COVID-19 group. This response was highly positive, considering that *Zheng et al.* (Zheng et al., 2020) pointed out that SARS-CoV-2 infection can lead to persistent autonomic dysfunction. Autonomic dysfunction is closely related to blood pressure control and, when altered, can result in unwanted increases in blood pressure levels. *Libby et al.* (Libby and Lüscher, 2021) found evidence of significant endothelial damage in patients with COVID-19, even in mild to moderate disease cases. The vascular endothelium plays a crucial role in regulating blood pressure, and any dysfunction in this layer of cells can lead to an imbalance in vascular homeostasis and ultimately contribute to the development of high blood pressure following COVID-19 infection (Libby and Lüscher, 2021). This finding suggests that ongoing assessment of cardiovascular health in patients recovered from COVID-19 is crucial, even in mild cases of the disease. Regular physical exercise has a proven and consistent acute and chronic hypotensive effect in normotensive and hypertensive individuals (Pescatello et al., 2004; Vona et al., 2009; Cornelissen and Smart, 2013). Several mechanisms are involved in this effect, including peripheral vasodilation, modulation of the autonomic nervous system, the release of nitric oxide, and reduction of oxidative stress and inflammation (Pescatello et al., 2004; Vona et al., 2009; Cornelissen and Smart, 2013). *Lemos et al.* (Lemos et al., 2022) identified higher DBP responses after 15 min of the Bruce test in hospitalized post-COVID-19 patients, a factor that suggests a sequel that involves endothelial damage and inflammatory responses, but the significant reduction observed in the moderate COVID-19 group takes the positive effects of physical exercise to promote non-medicamental treatment.

Another point that deserves attention is the self-reported symptoms with higher prevalence by the volunteers: fatigue (severe: 85.7%; moderate: 64.7%), muscle and joint pain (severe: 57.1%; moderate: 29.4%), and dyspnea (severe: 42.8%; moderate: 35.3%). Given this, independently of the COVID-19 severity disease, the sequels’ monitoring should be indispensable to reduce possible health impacts on the survivors.

Some biochemical analyses showed no significant changes after intervention: HbA1C, LDL-c, ALT, AST, ALP, and GAMMA-GT. However, the patient’s biochemical analyses were among the normative values in the pre-intervention time (Williamson and Synder, 2013). Significant changes were verified after intervention for CRP, albumin, fasting glucose, total cholesterol, HDL-c, and TGL.

CRP is one of the biomarkers associated with the severity of COVID-19 (although it is a nonspecific inflammation biomarker), and elevated levels are observed in hospitalized COVID-19 patients, especially those with severe disease (Guan et al., 2020), generating a systemic inflammatory response and increasing the release of pro-inflammatory cytokines. In pre-intervention, a high concentration of CRP was observed in the experimental groups compared with reference values (Aguiar et al., 2013), corroborating a previous study that revealed a high concentration of CRP in patients after recovery from COVID-19 (Ali et al., 2021). After the intervention, the CRP concentration was significantly reduced in the severe and moderate COVID-19 groups and the control group in response to multi-professional intervention, a factor that reinforces the effectiveness of physical exercise in reversing the inflammatory process (Improta-Caria et al., 2021).

The albumin levels at the beginning of the intervention within the values ​​considered as reference (3.5–4.8 d/dL) (Williamson and Synder, 2013); these findings are significant because, according to *Ali et al.* (Ali et al., 2021), hypoalbuminemia is seen in hospitalized COVID-19 patients, and this condition may persist after recovery and hospital discharge.

Following American Diabetes Association (Colberg et al., 2016), physical exercise is essential to control blood glucose in pre-diabetes and diabetes mellitus. The average values of the severe COVID-19 group in pre-intervention were classified in pre-diabetes, i.e., >100 mg/dL (Colberg et al., 2016) (pre: 101.4 ± 15.8 mg/dL and post: 96.1 ± 11.7; Δ = −4.5%). Despite no significant differences being observed for fasting glucose in the severe group, the relative delta reduction is positive since *Chourasia et al.* (Chourasia et al., 2023) pointed out aspects concatenated with diabetes post-COVID-19, linked with i) undiagnosed diabetes mellitus ii) SARS-CoV-2 virus affecting the pancreas and iii) hyperglycemia due to stress from acute COVID-19 infection that are associated with the disease severity. However, the moderate COVID-19 and control groups significantly reduced after the intervention. This response could be related to low-volume training (400 kcals/week) that increases insulin sensitivity in sedentary individuals (Dube et al., 2012).

The total cholesterol was significantly reduced for the control group after the intervention. Similar responses were identified after 8 weeks of concurrent training in untrained men (Ghahramanloo et al., 2009). The absence of differences between the moderate and severe COVID-19 groups could be explained because patients with COVID-19 may experience dysregulation of lipid profiles after COVID-19 (Zhao et al., 2022). Another point related to hospitalized COVID-19 patients is linked with low values of HDL-c during hospitalization and after discharge (Sampedro-Nuñez et al., 2021). Given this, considering the reduction of inflammation, healthy nutrition stimulus, and physical training intervention, the increased HDL-c in the severe COVID-19 group could be justified by the environmental, pathological, and physiological changes. Finally, the reduction of serum TGL is related to concurrent exercise stimulus at low to moderate intensity that promotes a considerable oxidation of this energetic substrate (Melzer, 2011).

A limitation of our study is the short-intervention period, which needed more to generate metabolic and physiological stress to produce significant changes in body composition and improvements in physical fitness, mainly in the severe COVID-19 group. Another limitation is lost follow-up among groups because the participants did not return for the final evaluations to perform a possible intent-to-treat analysis. Finally, consider the third limitation of the high drop-out rate among participants in our study, which promoted a β value lower than 80% for several analyses. Considering these responses, the findings observed in this article should be analyzed with caution and not be extrapolated for other spheres but could drive future multi-professional interventions. The high drop-out rate in longitudinal interventions is typical in Brazil since the study participants did not have financial support from the researchers. Many people needed to return to work after the governmental resources were finished. Thus, the patients returned to work, even with sequelae post-COVID-19.

To the best of these authors’ knowledge, this is the first study to consider the effect of multi-professional interventions according to symptomatology with an additional control group. Furthermore, this is the first study to enroll volunteers in a complete assessment of body composition and health-related physical fitness tests, including specific muscle strength tests, cardiorespiratory fitness tests, and biochemical markers. It is noteworthy that despite the absence of effects on body composition, the present maintenance of the measured variables is of great value given the vicious cycle of physical inactivity and the deleterious effects of lack of muscle contraction. The strengths of this study are physical exercise (concurrent training) combined with a multi-professional program, which can help patients return to their families, society, and work. In addition, the present study highlights the importance of a multi-professional team for recovering the overall health conditions of those who contracted COVID-19.

### 4.1 Final considerations

In short, the study’s design (clinical trial) with patients with different COVID-19 symptoms gives greater validity and a broader perspective of the effects of a physical exercise program enriched with nutritional education and psychoeducation in people with overweight and obesity. This study emphasizes the importance of developing strategies to recover health conditions through physical exercise, nutrition, and psychoeducation in COVID-19 survivors. Two months of concurrent training performed at a moderate intensity (according to the RPE scale) promoted significant improvements in lower limb muscle strength, increased albumin concentration, and significantly reduced inflammation in patients after hospital discharge for COVID-19. However, caution is needed since the hospitalized patients did not improve body composition and cardiorespiratory fitness, which suggests that long-term interventions are needed for COVID-19 survivors, especially for severe cases. Notably, COVID-19 responses were heterogeneous, and the biological individuality of each subject must be respected. Therefore, this study provides a concurrent training model that can be safely used in individuals affected by COVID-19 who wish to start a physical exercise program regardless of symptomatology and reinforces the importance of a multi-professional team with other health professionals, thereby seeking the individual’s integral care and support.

The severity of COVID-19 is a factor that limits the progression of the physical exercise program. Another point to consider is the various sequelae observed in individuals affected by COVID-19, which implies the need for individualized training based on the most sensitive observed difficulties during the initial physical evaluation. Thus, when considering concurrent training as part of a multi-professional program, professionals who provide exercise prescriptions should complete physical fitness assessments, that is, body composition and physical tests, and if possible, check the individual’s most recent blood test results. Concurrent training, proposed in this research, is a complete training model that stimulates cardiorespiratory and musculoskeletal fitness, thereby promoting improvements in physical fitness.

## Data Availability

The raw data supporting the conclusion of this article will be made available by the authors, without undue reservation.
